# Prospective Acceptability and Usability of a WhatsApp Chatbot for Epidemic Response in Malawi: Convergent Mixed Methods Study

**DOI:** 10.2196/83397

**Published:** 2026-10-28

**Authors:** Selemani Ngwira, Tsung-Shu Joseph Wu, Isaach Ndemera, Alanna Denny, Hsin-yi Lee, Griphin Baxter Chirambo, Ben Chilima, Matthew Kagoli, Yu Ting Sunny Hsieh, Balwani Mbakaya, Joseph Kwong Leung Yu, John O'Donoghue

**Affiliations:** 1 Research Department, Luke International, Plot No. KA/39, Katoto 2, Mzuzu, Malawi, 265 999000925; 2 Public Health Institute of Malawi, Lilongwe, Malawi; 3 Department of Overseas Mission, Pingtung Christian Hospital, Pingtung, Taiwan; 4 Department of Medicine & Health, School of Public Health,, University of College Cork, Cork, Ireland; 5 Nursing and Midwifery Department, Faculty of Health Sciences, Mzuzu University, Mzuzu, Malawi; 6 Graduate Institute of Environmental Engineering, National Taiwan University, Taipei, Taiwan; 7 Department of Public Health, University of Livingstonia, Mzuzu,, Malawi; 8 School of Medicine, University College Dublin, Dublin, Ireland; 9 ASSERT Research Centre, University College Cork, Cork, Ireland; 10 Malawi eHealth Research Centre, Mzuzu University, Mzuzu, Malawi; 11 Business Information Systems, University College Cork, Cork, Ireland

**Keywords:** digital health, sub-Saharan Africa, pandemic preparedness, chatbot, technology acceptance, WhatsApp

## Abstract

**Background:**

Digital health tools were widely adopted during the COVID-19 pandemic and helped to strengthen surveillance, risk communication, and health education. The government of Malawi launched the Malawi COVID-19 WhatsApp Chatbot on May 29, 2020. It remains unclear whether tools like this are acceptable and usable enough to warrant deployment in future epidemic responses.

**Objective:**

We assessed the prospective acceptability and usability of the Malawi COVID-19 WhatsApp Chatbot for future epidemic or pandemic preparedness and response.

**Methods:**

We used a convergent mixed methods design at a national workshop held from September 9 to 10, 2024, in Dowa District, Malawi. Thirty-nine participants (18 health workers and 21 members of the public) were purposively sampled from 10 districts. Quantitative and qualitative data were collected using the technology acceptance model (TAM) and Mobile Application Rating Scale (MARS) tools. Qualitative data from facilitated group discussions were analyzed with thematic analysis, using a hybrid deductive-inductive approach. We compared TAM scores (nonnormally distributed; Wilcoxon rank-sum test) and MARS scores (approximately normally distributed; independent-samples 1-tailed *t* test) between health workers and the public. Quantitative and qualitative findings were integrated at the interpretation stage.

**Results:**

Thirty-nine participants completed the TAM survey, and 37 completed the MARS survey. The mean overall TAM score was 4.33 (SD 0.82) out of 5, and the mean overall MARS score was 3.84 (SD 0.74) out of 5, both above each tool’s acceptability threshold of 3. Health workers scored the chatbot significantly higher than the public in the TAM (median difference 1.50, 95% CI 1.00‐2.00; *P*<.001) and MARS surveys (mean 4.03, SD 0.38 vs mean 3.71, SD 0.53; *P*=.04); no individual MARS subdomain showed a significant between-group difference. Among the participants, 80% said that they would use the chatbot again. Four themes emerged: accessibility and availability; awareness and acceptance; usage; and areas for improvement. Integration of the 2 strands suggested that the quantitative acceptance gap between groups was driven substantially by unequal prior awareness and exposure.

**Conclusions:**

Among this purposively selected group, the chatbot was viewed as prospectively acceptable and usable for future epidemic response. These findings indicate prospective support among engaged stakeholders and suggest that future investment should prioritize multilingual design and broader public awareness of digital tools.

## Introduction

Digital health innovations have demonstrated significant potential to strengthen public health surveillance, epidemic and pandemic preparedness, and response [1,2]. Countries with constrained testing and surveillance capacity, mostly across the African region, faced difficulty in controlling the COVID-19 pandemic [3,4]. The COVID-19 pandemic stretched the health systems of many countries across the globe, exposing major shortages in health workforce capacity and surveillance infrastructure.

Malawi’s health workforce grew by 32.7% over the previous 5 years. Despite this progress, a national workforce vacancy rate of 33% remains, limiting surge capacity during epidemic responses [1]. This gap in human resources affects the workforce during an epidemic or pandemic. The COVID-19 pandemic response witnessed insufficient staffing [2]. Digital health tools could aid in closing the gap, especially in surveillance and health education. The Malawi government is implementing the Malawi Healthcare Information System (MaHIS). This system is to manage electronic patient records in one central location [1]. Digital health innovations such as the MaHIS provide infrastructure that can be leveraged for surveillance and epidemic response during infectious disease outbreaks.

Malawi recorded 88,084 cases and 2683 deaths of COVID-19 as of October 2022, with the burden concentrated in its 3 city districts (Multimedia Appendix 1) [5]. Against this backdrop of constrained surveillance capacity, digital health systems, including WhatsApp (Meta Platforms, Inc) chatbots, WhatsApp-based reporting forums, and the One Health Surveillance Platform (OHSP) mode built on District Health Information System 2 (DHIS2; University of Oslo and the University of the Western Cape), were adopted in Malawi to extend the reach of case detection, public communication, and health education [3,6].

WhatsApp chatbots have been positioned as a low-cost, scalable channel for 2-way communication between health systems and the public. During outbreaks, it offers confidentiality and round-the-clock access to health information [4]. Evidence from low- and middle-income settings suggests that chatbot and conversational-agent acceptance is shaped strongly by language availability, digital literacy, and trust in the deploying institution [7-9]. However, evidence on how such tools are perceived by the health workers and the public remains limited, particularly in sub-Saharan African contexts where both populations may have starkly different digital access and prior exposure to the tool [10].

While the chatbot’s usage patterns during the pandemic have been described elsewhere [11], it remains unclear whether it can be considered acceptable and usable enough to warrant deployment in future epidemic responses. This question is distinct from, and arguably more policy-relevant than, asking how well the chatbot performed in specific circumstances, for example, the COVID-19 pandemic era: digital health investment decisions look forward to a tool’s value substantially in whether the people who used it would support its continued or expanded use.

This study addressed that gap. During a national workshop convened from September 9 to 10, 2024, we assessed the prospective acceptability and usability of the Malawi COVID-19 WhatsApp Chatbot for future epidemics and pandemics among health workers involved in the COVID-19 response and members of the public.

## Methods

### Study Design

This study used a convergent mixed methods design. Quantitative and qualitative data were collected concurrently and independently during a single national workshop, analyzed separately, and then merged for comparison and interpretation [12].

### Study Setting

The national workshop was held over one and a half days (September 9 and 10, 2024) at the Chiko Hotel in Mponela, Dowa District, Malawi, which served as the central venue for all participants.

The Malawi COVID-19 WhatsApp Chatbot was launched by the Ministry of Health (MoH) on May 29, 2020, under the leadership of the Digital Health Division (DHD). The chatbot enables the public to self-report suspected COVID-19 cases, access vaccination information, and view COVID-19 statistics. It operates on the Ministry’s official, Meta-certified WhatsApp number (+265-990-800-000), giving users seamless, verifiable access to the service. It was designed to be flexible and adaptable, allowing new information or services to be incorporated over time and extending its relevance beyond its original launch content.

### Participants, Sample Size, and Sampling

The study population comprised 2 groups: health workers involved in the COVID-19 response and members of the public affiliated with community-based organizations (CBOs). Participants were drawn from 10 districts of Lilongwe, Neno, Zomba, Mwanza, Ntchisi, Rumphi, Mzimba South, Mzimba North, Likoma, and Blantyre (Figure 1).

**Figure 1.**
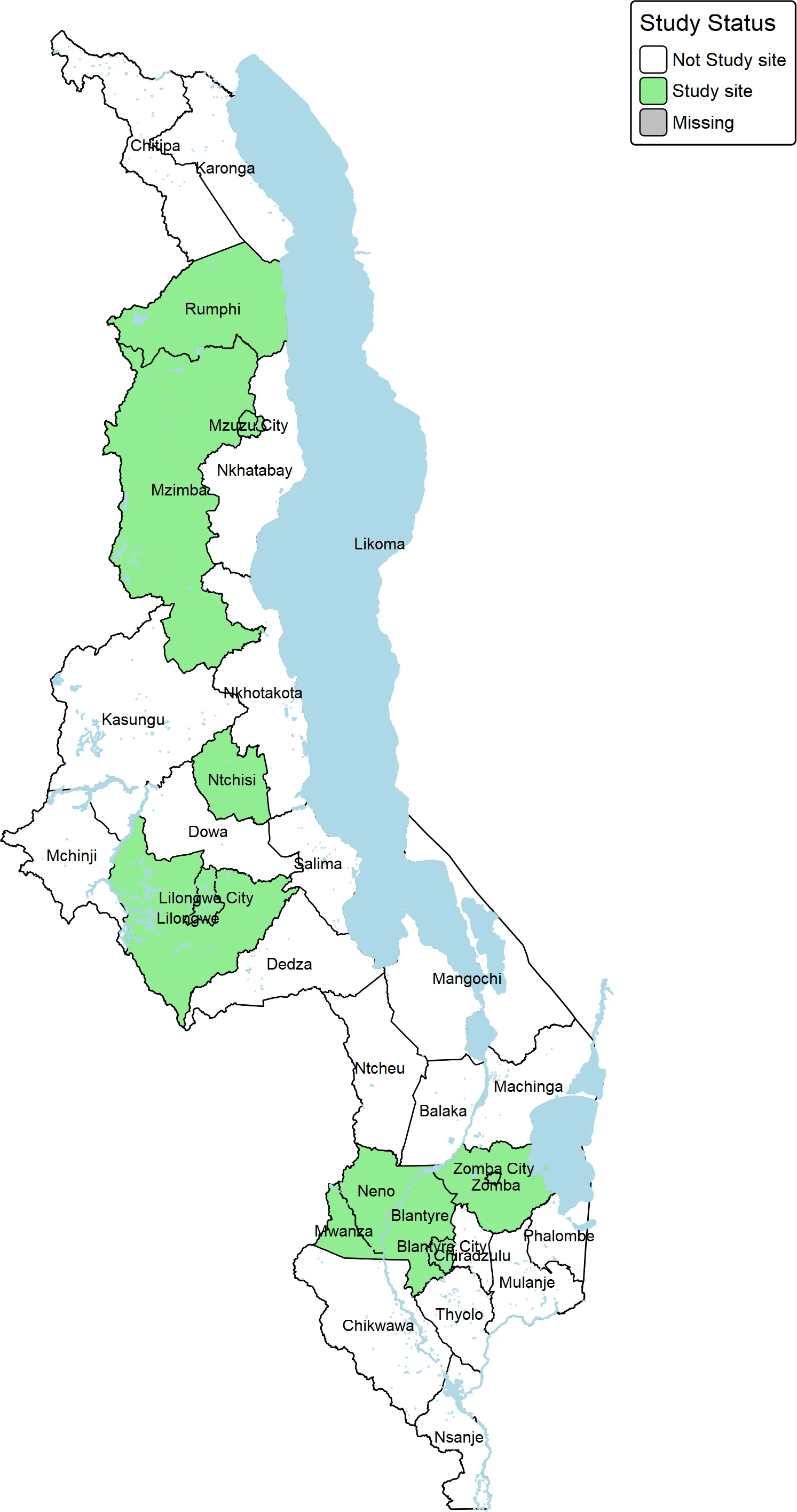
Study sites.

A total of 39 participants were purposively sampled (18 health workers and 21 members of the public). The sample size of 39 reflected the logistical capacity of a single national workshop, and participants were selected for their direct personal experience in the COVID-19 pandemic response and their prior use of digital health innovations.

Health workers were identified and approached through their respective District Health Offices. Members of the public were identified and approached through Malawi’s National Community-Based Organization Association. All participants were required to be available and willing to travel from their districts of residence to the national workshop site.

### Inclusion and Exclusion Criteria

Eligible participants were aged 18 years or older and had previously interacted with the Malawi COVID-19 WhatsApp Chatbot. For members of the public, eligibility additionally required affiliation with a CBO in one of the 10 selected districts. Individuals who had not previously interacted with the chatbot or who could not travel to the national workshop site were not eligible to participate.

### Data Collection

Quantitative data were collected using modified versions of the technology acceptance model (TAM) questionnaire and the Mobile Application Rating Scale (MARS), administered electronically using KoboCollect (version 2024.1.3; Kobo Tool box Organization) [13,14]. The TAM and MARS tools were modified to align with the Malawi COVID-19 chatbot context from the original validated wording. The tools were used to assess prospective perceptions of the Malawi COVID-19 WhatsApp Chatbot for future epidemics.

The TAM was used because the tool assesses technology acceptance. In this case, we were assessing the prospective willingness of a tool like a chatbot to be used in future epidemics in Malawi. The MARS tool was used in this study to assess the features of the Malawi COVID-19 WhatsApp Chatbot, with a prospective of which features can be adopted for a future app like the WhatsApp Chatbot.

The TAM tool was used to collect survey data during the workshop to measure how participants rated the chatbot. Participants were asked to complete it before the workshop activities started. A qualitative guiding question tool was structured using the constructs of the TAM tool to guide the group discussion.

The MARS tool was used to collect quantitative data. Qualitative data were collected through facilitated group discussions guided by a discussion guide developed from TAM constructs. Discussions were audio-recorded using an iPhone (Apple) voice recorder and supplemented with handwritten field notes before transcription using Express Scribe Transcription Software Pro (version 13.24; NCH software PTY limited).

### Data Analysis

#### Quantitative Data

TAM scores were calculated by summing scores across the tool’s 4 sections (perceived usefulness, perceived ease of use, attitude toward the chatbot, and behavioral intention to use the chatbot) and dividing by the number of sections, producing a score ranging from 1 to 5; a score of 3 or above indicated that the chatbot was “acceptable or adoptable.”

We conducted a subgroup analysis of TAM and MARS scores between health workers and the general population. This was done to assess differences in scoring between health workers and the general population due to different exposure to digital health tools. Normality of TAM and MARS scores was assessed using the Shapiro-Wilk test. TAM scores were not normally distributed; therefore, the nonparametric Wilcoxon rank-sum test (equivalent to the Mann-Whitney *U* test) was used for between-group comparisons, and medians are reported. MARS scores approximated a normal distribution; therefore, the parametric independent-samples 1-tailed *t* test was used for between-group comparisons, and means are reported.

MARS scores were calculated as the median across all 6 sections of the modified tool: the 4 standard objective quality sections (engagement, functionality, aesthetics, and information quality), the app subjective quality section, and the study-specific app-specific section addressing prospective health-behavior impact. Although the original MARS validation reports the objective quality subscales, subjective quality, and app-specific items as separate scores, we combined all 6 sections into a single overall score to provide one summary indicator of prospective chatbot quality and impact for this study. The objective, subjective, and the app-specific subscales were all distinct metrics; each section score is itself bounded between 1 and 5, so the resulting overall median remains on a 1-to-5 scale regardless of the number of sections averaged, producing a total median score out of 5; a median score of 3 or above indicated “acceptability,” and a score of 4 or above indicated “good” quality. All quantitative analyses were performed in R software (version 4.4.2; R Foundation for Statistical Computing).

#### Qualitative Data

Transcripts of the recorded group discussions, together with session notes, were analyzed using codebook thematic analysis, informed by Braun and Clarke’s [15] approach to thematic analysis using a hybrid deductive-inductive coding strategy. Two researchers (SN and IN) independently coded the transcripts. Coding followed a structured, line-by-line workflow: each researcher first read every transcript in full for familiarization, then reread each transcript line-by-line to inductively generate initial codes; these initial codes were compiled into a shared codebook of code labels and working definitions, which each researcher then applied and refined while coding independently. The 2 researchers came up with different themes based on codes that each had developed from the transcripts. The group discussion guide, structured around the MARS toolkit (Multimedia Appendix 2), provided a broad deductive organizing frame, while codes and themes were derived inductively from participants’ own responses, allowing patterns to emerge from the data within that structure.

A third researcher (AD) reviewed the coding and facilitated consensus on the final set of themes. Consensus was reached by voting, with all 3 researchers voting on the themes merged by AD from those developed by SN and IN. Coding was done manually in a spreadsheet, and Express Scribe was used only for transcription.

#### Integration

Consistent with the convergent mixed methods design, the quantitative and qualitative components were analyzed independently and subsequently merged at the interpretation stage. Quantitative TAM and MARS scores and qualitative themes addressing the same underlying construct were compared.

### Research Team and Reflexivity

The group discussions were facilitated by 2 members of the research team, SN and IN, both male research officers employed by Luke International and holding a Bachelor of Science degree at the time of the study. IN’s research focus is on qualitative methods, and he led the qualitative data collection and analysis; SN coordinated overall data collection and management. Neither facilitator had a prior personal or professional relationship with the participants before the workshop. Participants were informed about the study prior to the workshop through their respective institutions (for health workers) and through the CBO Association (for members of the general public) and were informed that the facilitators were researchers conducting an independent academic evaluation of the chatbot, not affiliated with the team that originally developed or operated it.

### Ethical Considerations

This study was reviewed and approved by the Mzuzu University Research Ethics Committee (reference number: MZUNIREC/DOR/24/76). Written informed consent was obtained from all participants before data collection. Participants were assigned a study subject ID by the research officer. All data were anonymized. Study subjects were compensated with US $10. The participants were also refunded transport and supported with a daily subsistence allowance.

## Results

Of the 39 participants enrolled in the workshop, all 39 completed the TAM preworkshop survey, and 37 completed the modified MARS survey. Two participants had missing MARS responses and were excluded from the MARS analyses. The TAM analyses reported in this study use the preworkshop assessment, which all participants completed.

### Participant Characteristics

Table 1 summarizes the sociodemographic characteristics of participants. The median age was 45 (IQR 34‐49) years; among the 37 participants, 30 (76.9%) were male and 7 (17.9%) female (2 missing). Members of the public made up 53.8% (n=21) of participants, and health workers made up 46.2% (n=18). The median years of experience responding to COVID-19 were 4 (IQR 3‐4) years. By occupation, 48.7% (n=18) of all participants were health workers, followed by farmers (n=5, 13.8%), business personnel (n=4, 10.8%), and fishermen and social workers (n=3, 8.1% each), with the remainder spread across community organization leaders, housewives, retired civil servants, and other occupations. Among health worker participants specifically, the largest group worked in surveillance (9/18, 50%), followed by smaller numbers from the Emergency Operation Centre (EOC), laboratory, DHD, and one each from coordination and resource mobilization, Expanded Program on Immunization (EPI), and Risk Communication and Community Engagement (RCCE).

**Table 1.** Background characteristics of social and demographic variables.

Characteristic	Frequency
Participant category, n (%)	
Member of the public	21 (53.8)
Health worker	18 (46.2)
Age (years), median (IQR)	45 (34-49)
Sex, n (%)	
Female	7 (17.9)
Male	30 (76.9)
Not recorded^a^	2 (5.1)
District, n (%)	
Blantyre	4 (10.3)
Likoma	3 (7.7)
Lilongwe	6 (15.4)
Mwanza	3 (7.7)
Mzimba North	2 (5.1)
Mzimba South	5 (12.8)
Neno	5 (12.8)
Ntchisi	6 (15.4)
Rumphi	3 (7.7)
Zomba	2 (5.1)
COVID-19 response pillar^b^, n (%)	
CMED^c^/ coordination and resource mobilization	1 (5.6)
Digital Health Division	2 (11.1)
Emergency Operation Centre	2 (11.1)
EPI^d^	1 (5.6)
Laboratory	2 (11.1)
Risk communication and community engagement	1 (5.6)
Surveillance	9 (50)
Experience in COVID-19 response (years), median (IQR)	4 (3-4)
Occupation designation, n (%)	
Business	4 (10.8)
CSO^e^ leader	1 (2.7)
Farmer	6 (16.2)
Fisherman	3 (8.1)
Health worker	18 (48.6)
Housewife	2 (5.4)
Other	1 (2.7)
Retired	1 (2.7)
Social worker	3 (8.1)

a The missing data within the gender variable were due to undocumented responses during data collection.

b Breakdown applies only to the 18 health worker participants, not the full (n=39).

c CMED: Central Monitoring and Evaluation Division.

d EPI: Expanded Program on Immunization.

e CSO: civil society organization.

### Quantitative Findings: Acceptance of the Chatbot (TAM)

The overall TAM acceptance mean score was 4.33 (SD 0.82) out of 5, above the threshold of 3. Scores were similarly high across all 4 TAM subdomains: perceived usefulness (mean 3.69, SD 0.85), perceived ease of use (mean 4.27, SD 0.86), attitude toward the chatbot (mean 4.23, SD 0.83), and behavioral intention to use the chatbot (mean 4.19, SD 0.81). TAM’s behavioral intention subdomain is prospective. The score indicates participants’ prospective willingness to use the chatbot, or a similar tool, in a future epidemic or pandemic.

Acceptance differed by participant group: health workers were significantly more likely than members of the public to find the chatbot acceptable overall (Wilcoxon rank-sum test; median difference [MD] 1.50, 95% CI 1.00‐2.00; *P*<.001), as well as on each subdomain perceived usefulness (MD 1.50; *P*<.001), perceived ease of use (MD 1.50; *P*<.001), attitude (MD 1.00; *P*=.004), and behavioral intention (MD 1.00; *P*=.004; Table 2). This gap is examined further alongside the qualitative findings, in which lower public awareness emerges as a plausible contributor.

**Table 2.** The difference between health workers at the Ministry of Health (MoH) and members of the public regarding acceptance of the Malawi WhatsApp COVID-19 chatbot.

TAM^a^ score variable	MoH, median score (IQR)	MoH, mean score (SD)	General public, median score (IQR)	General public, mean score (SD)	Median difference (95% CI)	Mean difference
Overall total score	5 (5‐5)	4.9 (0.35)	4 (3‐4)	3.9 (0.86)	1.50 (1.00-2.00)	1
Perceived usefulness	4 (4‐5)	4.5 (0.51)	3 (3‐3)	3.1 (0.49)	1.50 (1.50-2.00)	1.4
Perceived ease of use	5 (5‐5)	4.8 (0.38)	4 (3‐4)	3.8 (0.87)	1.50 (1.00-1.50)	1
Attitude toward chatbot	5 (4‐5)	4.6 (0.49)	4 (3‐5)	3.9 (0.96)	1.00 (0.50-2.00)	0.7
Behavior and intention of the chatbot	5 (4‐5)	4.6 (0.49)	4 (4‐5)	3.8 (0.90)	1.00 (0.50-1.50)	0.8

a TAM: technology acceptance model.

Participants reported an overall mean of 1.77 (SD 1.58) years of direct prior experience using the chatbot (mean 2.67, SD 1.27 y among MoH, mean 0.47, SD 0.92 y among general public). Among the participants, 80% indicated they would use the chatbot in a future epidemic. A result that is consistent with the high TAM behavioral-intention scores above and indicating broad prospective support for the tool’s continued or expanded use.

### Quantitative Findings: Usability and Quality (MARS)

The overall MARS score was 3.84 (SD 0.74) out of 5, above the threshold of 3, though below the threshold of 4 used to indicate “good” quality. Functionality was the most highly rated domain (mean 4.03, SD 0.76), suggesting that participants found the chatbot’s core features worked as intended; engagement was the least highly rated (mean 3.62, SD 0.64; Figure 2), suggesting more limited success in sustaining user interest or interaction over time, a pattern that aligns with the qualitative theme of underutilization.

**Figure 2.**
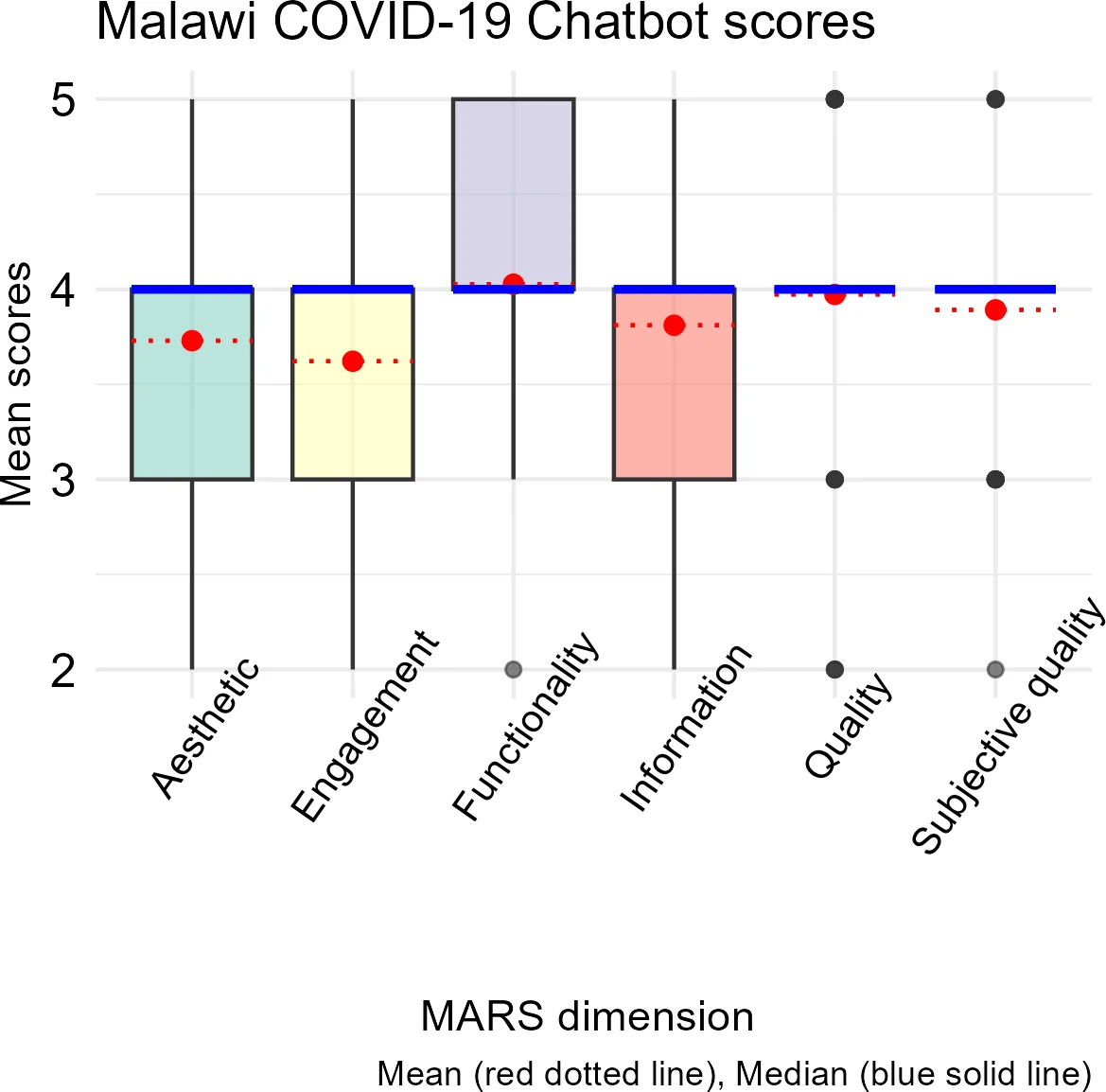
Overall Mobile Application Rating Scale (MARS) rating.

Health workers rated the chatbot significantly more highly overall (mean 4.03, SD 0.38) than members of the public (mean 3.71, SD 0.53; independent-samples 1-tail *t* test, *P*=.04; Table 3). However, no individual MARS sections showed a statistically significant difference between the 2 groups, suggesting that the overall gap reflects a general difference toward the tool rather than a sharply localized disagreement about any one feature.

**Table 3.** MARS^a^ scores by participant category.

MARS section	MoH^b^, mean (SD)	General public, mean (SD)
Overall score	4.03 (0.38)	3.71 (0.53)
Engagement	3.67 (0.62)	3.59 (0.67)
Functionality	4.40 (0.74)	3.77 (0.69)
Aesthetic	3.80 (0.77)	3.68 (0.72)
Information	4.07 (0.70)	3.64 (0.79)
Subjective quality	3.93 (0.59)	3.86 (0.64)

a MARS: Mobile Application Rating Scale.

b MoH: Ministry of Health, denoting health workers.

### Qualitative Findings

Four themes emerged from the group discussions: accessibility and availability, awareness and acceptance, usage, and areas for improvement.

#### Theme 1: Accessibility and Availability

Participants described 2 structural barriers limiting who could access the chatbot, independent of attitudes toward the tool itself: language coverage and smartphone ownership. The chatbot was available only in Chichewa and English, which participants noted disadvantaged speakers of other languages.

*[T]here were only two languages, Chichewa and English, and my colleagues from North could have challenges with the Languages…*

Smartphone ownership was identified as a further constraint, since the chatbot required a smartphone for access:

*We know about the people in the village; most do not have smartphones.*

Participants also noted an offsetting advantage: because the chatbot operated over WhatsApp, it required only the standard data bundle that most community members with smartphones could already afford, lowering the marginal cost of access for that group.

*Someone who has a WhatsApp data bundle will be able to use the system, so it was already an opportunity.*

These accounts suggest that reach was the primary obstacle for community members without a smartphone or without proficiency in Chichewa or English, a distinction that matters for future implementation since it points to a specific design.

A further accessibility barrier was technical: participants reported that selecting the Chichewa language option sometimes failed silently, without an error message, whereas the English version displayed a clear error message when something went wrong. Participants described this as personally; repeated unexplained failures led some users to conclude they themselves were doing something wrong, prompting them to stop using the chatbot altogether.

*When one selects Chichewa, it shows that something’s wrong, so when you take a person and technical reality, it gives a sense like I am the one who made that mistake. Doing two to three times, you concluded that I am the one making the mistake, hence I will not use this app again.*

This account points to a specific, fixable technical gap in consistent error handling and messaging across both language options, rather than to a deeper barrier in language coverage or device access.

#### Theme 2: Awareness and Acceptance

Participants linked low awareness of the chatbot among community members to the underutilization of existing community communication channels, particularly CBOs:

*[F]or the messages to reach a large number of community members, it is important to use CBOs.*

Some participants reported first hearing about the chatbot at the workshop itself, having previously received COVID-19 information through other channels, such as radio:

Chatbot *is a new word to me; this is my first time hearing about it. All messages about COVID-19 were passed through radio stations. CBO got the information regarding COVID-19 through the radio.*

*The chatbot for us CBOs, we need to understand clearly if there can be an opportunity specific to discuss and talk on the chatbot with the CBOs, because this information we did not have.*

This theme offers a plausible explanation for the quantitative gap reported above: lower TAM and MARS scores among the public likely reflect lower awareness and exposure, rather than a more negative assessment of the chatbot’s design or usefulness in a future epidemic.

Participants proposed specific enhancements: expanding the number of supported languages, adding infection-prevention content, extending the chatbot’s scope beyond COVID-19 to other epidemics, and adding the ability to recognize and respond to audio input.

*This app might not be limited to COVID-19; if possible, can it also take care of the other epidemics, emerging or reemerging infections?*

Participants gave specific examples of other diseases to which the chatbot’s scope could be extended, naming current outbreaks of concern rather than speaking only in general terms:

*[A]s a suggestion, can the app include some of the things like this time we are talking of Mpox and Cholera.*

Beyond the disease scope, participants requested additional content areas, specifically infection prevention and control, and case management information that the chatbot did not provide during the pandemic:

*Right now, the chatbot has COVID-19 statistic and vaccination, but we are thinking of other important areas that we feel were missed, things to do with infection prevention, or case management.*

One health worker proposed a more substantial technical enhancement: an AI-supported feature that could automatically identify the geographic origin of a reported issue and route it to the designated district-level responsible person for follow-up.

*[I]f we can embed an AI tool that can help to capture the place where the issues are coming.... You integrate a resource person from the district…will be following up and responding to them.*

Separately, several members of the public affiliated with CBOs argued that future digital health implementations should give CBOs a more central, decision-making role rather than a consultative one, citing their comparative cost-effectiveness and trusted standing in communities as reasons to lead rather than simply support future rollouts.

*The use of CBOs is cheap and very cost-effective and efficient.*

*Let the CBOs lead and participate in your decision-making.*

These suggestions directly inform the study’s prospective aim: rather than describing what participants think happened during COVID-19, they describe what participants want from a similar tool for future epidemic responses.

#### Theme 3: Usage

Participants described the chatbot as underutilized by the public during the pandemic, attributing this primarily to limited promotion rather than to the tool’s quality:

*[The app] has proven to be a good tool, but it did not receive the kind of attention that people can utilize it.*

This account is consistent with the MARS findings above, where engagement was the lowest-rated domain despite functionality being the highest-rated, suggesting that the chatbot’s core features were not the limiting factor but rather the extent to which people were made aware of and drawn into sustained use.

Participants also suggested broadening the chatbot’s delivery channels beyond WhatsApp, citing comparable services that use SMS to reach users without requiring a smartphone or app installation:

*[T]he same WhatsApp chatbot being created on other platforms that uses SMS...*

Separately, participants raised a methodological concern about the representativeness of the workshop’s own feedback: several noted that views gathered from this group risked being more theoretical than those of people who had used the chatbot frequently and suggested directly following up with heavy users in any future evaluation of digital tools used during an epidemic.

*I think riding on this comment and people who have truly used the application. is it possible for you to identify them and get feedback from them? Because you will going to get true feedback and unlike what we are going you. Will be more theoretical but those who have truly used the platform...*

### Integration of Quantitative and Qualitative Findings

The quantitative and qualitative findings were compared directly to assess where the 2 strands agree or diverge:

#### Acceptance Gap Between Groups

The quantitative finding that health workers scored significantly higher than the members of the public on both TAM and MARS aligns with the qualitative theme of awareness and acceptance, in which some members of the public reported first learning about the chatbot only at the workshop itself. This convergence suggests that the quantitative gap is driven substantially by unequal prior exposure.

#### Engagement vs Functionality

The quantitative finding that functionality was the highest-rated MARS domain while engagement was the lowest converges with the qualitative theme of usage, in which participants attributed underutilization to insufficient promotion rather than to problems with how the chatbot worked.

#### Reach as a Distinct Barrier

The qualitative theme of accessibility and availability (language coverage and smartphone ownership) identifies barriers to reach that are not captured by the TAM or MARS scores at all. This is a case of qualitative findings surfacing a constraint that the quantitative strand could not detect by design, rather than the 2 strands converging or diverging on the same construct.

## Discussion

### Principal Results

This study found that the Malawi COVID-19 WhatsApp Chatbot was prospectively acceptable and usable for future epidemic response among health workers and members of the public who had direct prior experience with the tool, with both the TAM and MARS scores exceeding each instrument’s respective acceptability threshold (see the Results section for full scores). For public health policy, this suggests that WhatsApp-based chatbots represent a viable, low-infrastructure channel for epidemic risk communication and surveillance support within Malawi’s health system. However, acceptance was not uniform: scores were consistently higher among health workers than among members of the public across both instruments, indicating that future deployment cannot assume equal readiness for uptake across occupational and community groups and that targeted strategies to build public familiarity and trust are likely necessary alongside any future rollout.

The findings reflect the views of a purposively selected, experienced group of respondents rather than the general population: 80% of participants indicated they would use the chatbot in future epidemics. Participants, particularly health workers, recommended broadening the disease scope beyond COVID-19. Taken together with the qualitative findings, this suggests cautious support among this sample for retaining and adapting chatbot-based tools for future epidemic and pandemic preparedness.

The qualitative findings also surfaced structural barriers independent of attitudes toward the chatbot itself: limited smartphone ownership among community members and the restriction of the chatbot to Chichewa and English, which participants noted disadvantaged speakers of other languages. These barriers describe constraints on reach, distinct from the acceptability findings from TAM or MARS, and point to specific, actionable improvements, such as language expansion and multichannel delivery.

### Comparison With Prior Work

Chatbots are increasingly used and accepted as a channel for health service delivery [16], a pattern consistent with the prospective acceptance found among health workers in this study. Health workers in this workshop viewed the Malawi COVID-19 WhatsApp Chatbot as a tool worth retaining for future use, consistent with broader reporting on chatbots’ perceived value in extending confidential, low-barrier access to health information without requiring an in-person facility visit [9,17].

Language availability is a recurring constraint on chatbot accessibility [9], and we find that the Chatbot was restricted to Chichewa and English; limited access for speakers of other local languages is consistent with that literature. An educational chatbot for people with hemophilia in Senegal was built in the local language (Wolof). The study findings suggest that a culturally adapted digital conversational agent is likely to be used by people with hemophilia in Senegal [17]. A culturally adapted digital tool is built upon local context. Language was one of the structural barriers to the use of generative AI [18]. Developing digital health innovations only in foreign languages, without local-language options, may further limit their reach among populations with lower literacy in those languages, reducing wider uptake.

Participants’ support for broadening the chatbot’s disease scope beyond COVID-19 aligns with arguments that digital surveillance and social media tools offer a practical foundation for preparedness and response for future outbreaks, not just the pandemic for which they were originally built [19]. This provides an opportunity to strengthen surveillance. The use of chatbots for future pandemics or epidemics is acceptable in countries like Malawi, and the acceptance of digital health surveillance in Malawi is acceptable to the public [8].

### Limitations

This study has several limitations that should inform the interpretation of the findings. First, the sample was not population-based: participants were purposively selected based on COVID-19 disease burden in their district and, for health workers, on direct involvement in the COVID-19 response, while members of the public were selected through affiliation with a CBO. This selection strategy means participants likely had greater-than-average awareness of, and engagement with, COVID-19 response structures compared with the general population; individuals without CBO affiliation, or health workers outside the invited pillars, may hold different views and were not captured. The convenience element of recruitment requires willingness and ability to travel to a single national workshop site, further selected for participants with fewer logistical or resource constraints, which may not represent those most affected by the access barriers identified in the qualitative findings.

Second, both health workers and members of the public reported limited direct interaction with the chatbot, with a mean experience of 1.74 (SD 1.58) years of exposure over a roughly 4-year period since deployment. Responses on usability and usefulness reflect relatively light, and in some cases indirect or second-hand, exposure to the tool rather than intensive routine use. This is distinct from and should not be conflated with a recall-validity concern about rating the chatbot’s pandemic-era performance: participants were asked about their prospective willingness to use and support such a tool in future epidemic response, a forward-looking judgment that does not require extensive historical use to be meaningfully reported, though it is informed by whatever direct experience participants had.

Third, the small sample size (n=39) and the qualitative data being collected from group discussions rather than individual interviews limit the depth of attributable individual perspectives and the generalizability of both the quantitative and qualitative findings beyond this group.

Fourth, participants in the group discussions themselves raised a related concern: that workshop-based feedback may not represent the views of individuals who had used the chatbot most frequently and recommended directly following up with these heavy users in any future evaluation. We did not identify or recruit specific heavy users of the chatbot platform because doing so would have required accessing personal user data (eg, contact information) held by the MoH, which Malawian data protection law does not permit researchers to obtain without the original users’ informed consent to such secondary use. This constraint, while methodologically limiting, reflects a deliberate decision to protect participant privacy rather than an oversight, and future studies wishing to capture heavy-user perspectives directly would need to build informed consent for research follow-up into the chatbot’s own terms of use from the outset.

Participants also identified a gap relative to the MARS evaluation framework itself: the chatbot had no entertainment or engagement-oriented features, a dimension the MARS tool explicitly assesses as part of sustaining user interest; this was a limitation of using the MARS tool to assess an app like the Malawi COVID-19 WhatsApp Chatbot and some 4 items of the original MARS toolkit were omitted because they were not in line with the context and the objective of the study. We further note that our reported overall MARS score combines all 6 sections of the modified tool—the 4 objective quality sections, the app subjective quality section, and the study-specific app-specific section—into a single mean. This departs from the original MARS validation, which reports objective quality, subjective quality, and app-specific items as distinct scores; we chose to combine them here to provide a single prospective summary indicator, and this should be considered when comparing our reported score against studies using standard MARS scoring conventions.

### Conclusions

The Malawi COVID-19 WhatsApp Chatbot was viewed as a prospectively acceptable and usable digital health tool for future epidemic and pandemic response, with higher acceptance among health workers than among members of the public. Participants identified specific, actionable barriers to broader uptake, limited language coverage, and reliance on smartphone ownership, alongside a shared interest in expanding the chatbot’s scope beyond COVID-19 to other epidemics.

These findings, drawn from a purposively selected group with above-average exposure to Malawi’s COVID-19 response structures, should be read as an indication of prospective support for chatbot-based tools among engaged stakeholders, rather than as evidence of the chatbot’s demonstrated impact during the pandemic itself. The study suggests that future investment in similar tools should prioritize multilingual design and strategies to raise awareness among the public, particularly community members without ties to existing organizational structures, if such tools are to achieve a broader reach in future epidemic preparedness and response.

## Supplementary material

10.2196/83397 Multimedia Appendix 1 Cumulative distribution of COVID-19 cases.

10.2196/83397 Multimedia Appendix 2 Mobile Application Rating Scale (MARS) tool.

## Abbreviations

CBO: community-based organization
DHD: Digital Health Division
DHSI2: District Health Information System 2
EOC: Emergency Operation Centre
EPI: Expanded Program on Immunization
MaHIS: Malawi Healthcare Information System
MARS: Mobile Application Rating Scale
MD: median difference
MoH: Ministry of Health
OHSP: One Health Surveillance Platform
RCCE: Risk Communication and Community Engagement
TAM: technology acceptance model

## References

[1] (2024). Health sector annual progress report for the 2023-24 fiscal year. https://health.gov.mw/storage/resources/Annual%20Report%202023-24%20FY_FINAL_06122024docx%201.pdf.

[2] Patel P, Adebisi YA, Steven M, Lucero-Prisno Iii DE (2020). Addressing COVID-19 in Malawi. Pan Afr Med J.

[3] Scott BK, Miller GT, Fonda SJ (2020). Advanced digital health technologies for COVID-19 and future emergencies. Telemed J E Health.

[4] Manirambona E, Khan NE, Ogungbe O (2024). Association between e-health literacy and perceived importance of future pandemic preparedness in sub-Saharan Africa. Sci Rep.

[5] Afuleni MK, Cahuantzi R, Lythgoe KA (2025). Epidemiological and phylogenetic analyses of public SARS-CoV-2 data from Malawi. PLoS Glob Public Health.

[6] Fagherazzi G, Goetzinger C, Rashid MA, Aguayo GA, Huiart L (2020). Digital health strategies to fight COVID-19 worldwide: challenges, recommendations, and a call for papers. J Med Internet Res.

[7] Cheah MH, Gan YN, Altice FL (2024). Testing the feasibility and acceptability of using an artificial intelligence chatbot to promote HIV testing and pre-exposure prophylaxis in Malaysia: mixed methods study. JMIR Hum Factors.

[8] Thawani A, Ngwira S, Masoambeta J (2026). Public acceptance of digital health surveillance tools in Malawi: evidence from a multi-district household survey. BMC Digit Health.

[9] Phiri M, Munoriyarwa A (2023). Health chatbots in Africa: scoping review. J Med Internet Res.

[10] Awosiku OV, Gbemisola IN, Oyediran OT (2025). Role of digital health technologies in improving health financing and universal health coverage in sub-Saharan Africa: a comprehensive narrative review. Front Digit Health.

[11] Ndemera I, Hsieh YTS, Chirwa K (2026). Describing a national chatbot deployed by the Ministry of Health in Malawi during the COVID-19 pandemic: retrospective data analysis. J Med Internet Res.

[12] Perreault K (2011). Research design: qualitative, quantitative, and mixed methods approaches. Man Ther.

[13] Azuddin NA, Manan KA, Abdul Ghani MT (2024). Developing questionnaires for the technology acceptance model (TAM): a study on the teachers’ acceptance of using online education. J Komunikasi Malays J Commun.

[14] Stoyanov SR, Hides L, Kavanagh DJ, Zelenko O, Tjondronegoro D, Mani M (2015). Mobile App Rating Scale: a new tool for assessing the quality of health mobile apps. JMIR mHealth uHealth.

[15] Braun V, Clarke V (2006). Using thematic analysis in psychology. Qual Res Psychol.

[16] Jovanovic M, Baez M, Casati F (2021). Chatbots as conversational healthcare services. IEEE Internet Comput.

[17] Babington-Ashaye A, de Moerloose P, Diop S, Geissbuhler A (2023). Design, development and usability of an educational AI chatbot for People with Haemophilia in Senegal. Haemophilia.

[18] Gondwe G (2023). CHATGPT and the Global South: how are journalists in sub-Saharan Africa engaging with generative AI?. Online Media Glob Commun.

[19] Kostkova P, Saigí-Rubió F, Eguia H (2021). Data and digital solutions to support surveillance strategies in the context of the COVID-19 pandemic. Front Digit Health.

