# Supplementary material for: Prospective Acceptability and Usability of a WhatsApp Chatbot for Epidemic Response in Malawi: Convergent Mixed Methods Study
Source: JMIR Form Res. 2026 Oct 28;10:e83397. doi: 10.2196/83397 (PMC13732754; doi:10.2196/83397)
Supplement: Supplementary file 1 [file formative-v10-e83397-s001.docx]

Multimedia Appendix

*Table 4: Cumulative Distribution of COVID-19 Disease Burden in Malawi*

| **District** | **Cases** | **AR** | **District** | **Cases** | **AR** |
| --- | --- | --- | --- | --- | --- |
| Blantyre | 24895 | 18.54 | Chitipa | 720 | 2.88 |
| Likoma | 148 | 9.52 | Chiradzulu | 1078 | 2.83 |
| Mzimba South | 2437 | 9.51 | Kasungu | 2432 | 2.65 |
| Lilongwe | 20684 | 7.34 | Mulanje | 1764 | 2.38 |
| Neno | 1042 | 7.01 | Mangochi | 2952 | 2.3 |
| Mzimba North | 5385 | 5.42 | Dowa | 1848 | 2.18 |
| Zomba | 4157 | 4.52 | Balaka | 1014 | 2.09 |
| Mwanza | 647 | 4.44 | Phalombe | 825 | 1.75 |
| Ntchisi | 1489 | 4.24 | Nsanje | 530 | 1.66 |
| Rumphi | 1038 | 4.21 | Dedza | 1372 | 1.53 |
| Karonga | 1563 | 3.98 | Chikwawa | 919 | 1.51 |
| Nkhatabay | 1110 | 3.67 | Ntcheu | 1043 | 1.44 |
| Mchinji | 2370 | 3.64 | Thyolo | 1085 | 1.41 |
| Salima | 1909 | 3.61 | Machinga | 1020 | 1.23 |
| Nkhotakota | 1439 | 3.39 |  |  |  |
