# Supplementary material for: Prospective Acceptability and Usability of a WhatsApp Chatbot for Epidemic Response in Malawi: Convergent Mixed Methods Study
Source: JMIR Form Res. 2026 Oct 28;10:e83397. doi: 10.2196/83397 (PMC13732754; doi:10.2196/83397)
Supplement: Supplementary file 2 [file formative-v10-e83397-s002.docx]

**Annex1: MARS tool kit.**

Mobile Application Rating Scale (MARS) for MoH WhatsApp COVID-19 ChatBot

Affiliations: □ General public □MoH

Age group (all that apply)□ Children (under 12) □ Adolescents (13-17) □ Young Adults (18-25) □ Adults

Technical aspects of app (all that apply)

□ Allows sharing information (Facebook, Twitter, What’sAapp uses etc.)

□ Sends reminders

□ Needs web access to function

App Quality Ratings

The rating scale assesses app quality using four dimensions. All items are rated on a 5-point scale from “1. Inadequate” to “5. Excellent”. Circle/select the number that most accurately represents the quality of the app component you are rating. Please use the descriptors provided for each response category.

SECTION A

Engagement – fun, interesting, customisable, interactive (e.g. sends alerts, messages, reminders, feedback, enables sharing), well-targeted to the audience

1. Entertainment: Is the COVID-19 WhatsApp Chatbot fun/entertaining to use? Does it use any strategies to increase engagement through entertainment?

1 Dull, not fun or entertaining at all

2 Mostly boring

3 OK, fun enough to entertain the user for a brief time (< 5 minutes)

4 Moderately fun and entertaining, would entertain user for some time (5-10 minutes total)

5 Highly entertaining and fun, would stimulate repeat use

2. Interest: Is the COVID-19 WhatsApp Chatbot enjoyable to use? Does it use any strategies to increase engagement by excitingly presenting its content?

1 Not interesting at all

2 Mostly uninteresting

3 OK, neither exciting nor uninteresting; would engage the user for a brief time (< 5 minutes)

4 Moderately interesting; would engage the user for some time (5-10 minutes total)

5 Very interesting, would engage the user in repeat use

3. Customisation: Does the COVID-19 WhatsApp Chatbot provide/retain all necessary /preferences features (e.g., content, notifications, updates, etc.)?

1 Does not allow any customisation or requires setting to be input every time

2 Allows insufficient customisation limiting functions

3 Allows basic customisation to function adequately

4 Allows numerous options for customisation

5 Allows complete tailoring to the individual’s characteristics/preferences, retains all settings

4. Interactivity: Does the COVID-19 WhatsApp Chatbot allow user input, provide feedback, and contain prompts (reminders, sharing options, notifications, etc.)?

1 No interactive features and/or no response to user interaction

2 Insufficient interactivity, or feedback, or user input options, limiting functions

3 Basic interactive features to function adequately

4 Offers a variety of interactive features/feedback/user input options

5 Very high level of responsiveness through interactive features/feedback/user input options

5. Target group: Is the COVID-19 WhatsApp Chatbot content (visual information, language, design) appropriate for its target audience?

1 Completely inappropriate/unclear/confusing

2 Mostly inappropriate/unclear/confusing

3 Acceptable but not targeted. It may be inappropriate/unclear/confusing

4 Well-targeted, with negligible issues

5 Perfectly targeted, no issues found

SECTION B

Functionality – app functioning, easy to learn, navigation, flow logic,

and gestural design of app

6. Performance: How accurately/fast do the COVID-19 WhatsApp Chatbot features (functions) and components (buttons/menus) work?

1, insufficient/inaccurate response (e.g. some prompts do not respond)

2, Some functions work but lagging or contain major technical problems

3, works overall. Some technical problems need fixing/Slow at times

4 Mostly functional with minor/negligible problems

5 Perfect/timely response; no technical bugs found/contains a ‘loading time left’ indicator

7. Ease of use: How easy is learning to use the COVID-19 WhatsApp Chatbot; how clear are the menu labels/icons and instructions?

1 No/limited instructions; menu labels/icons are confusing; complicated

2 Useable after a lot of time/effort

3 Useable after some time/effort

4 Easy to learn how to use the COVID-19 WhatsApp Chatbot

5 Able to use the COVID-19 WhatsApp Chatbot immediately; intuitive; simple

8. Navigation: Is moving between pages logical, accurate, appropriate, and uninterrupted? Are all necessary COVID-19 Information present?

1 Different sections within the app seem logically disconnected and random/confusing/navigation is difficult

2 Usable after a lot of time/effort

3 Usable after some time/effort

4 Easy to use or missing a negligible feature

5 Perfectly logical, easy, clear and intuitive pages flow throughout or offers shortcuts

9. Gestural design: Are interactions consistent and intuitive across all components/pages?

1 Completely inconsistent/confusing

2 Often inconsistent/confusing

3 OK with some inconsistencies/confusing elements

4 Mostly consistent/intuitive with negligible problems

5 Perfectly consistent and intuitive

SECTION C

Aesthetics – graphic design, overall visual appeal, colour scheme, and stylistic consistency

10. Layout: Is the arrangement and size of the page's icons/menus/content appropriate or zoomable if needed?

1 Very bad design, cluttered, some options impossible to select/locate/see/read device display not optimised

2 Bad design, random, unclear, some options challenging to select/locate/see/read

3 Satisfactory, few problems with selecting/locating/seeing/reading items or with minor screen size problems

4 Mostly clear, able to select/locate/see/read items

5 Professional, simple, clear, orderly, logically organised, device display optimised. Every design component has a purpose

11. Graphics: How high is the quality/resolution of graphics used for icons/menus/content?

1 Graphics appear, amateur, very poor visual design - disproportionate, completely stylistically inconsistent

2 Low-quality/low-resolution graphics; low-quality visual design – disproportionate, stylistically inconsistent

3 Moderate-quality graphics and visual design (generally consistent in style)

4 High-quality/resolution graphics and visual design – mostly proportionate, stylistically consistent

5 Very high-quality/resolution graphics and visual design - proportionate, stylistically consistent throughout

12. Visual appeal: How good does the COVID-19 chatbot content( font, Color and Size)?

1 No visual appeal, unpleasant to look at, poorly designed, clashing/mismatched colours/font and Size

2 Little visual appeal – poorly designed, bad use of colour/font/Size, visually boring

3 Some visual appeal – average, neither pleasant nor unpleasant

4 High level of visual appeal – seamless graphics – consistent and professionally designed

5 As above + very attractive, memorable, stands out; use of colour enhances app features/menus

SECTION D

Information – Contains high-quality information (e.g. text, feedback, measures, references) from a credible source. Select N/A if the app component is irrelevant.

13. Accuracy of the COVID-19 WhatsApp Chatbot description (on profile): Does the COVID-19 WhatsApp Chatbot profile contain its purpose?

1 Misleading. The chatbot profile does not contain the description components/functions. Or has no description 2 Inaccurate. The profile contains very few of the described components/functions

3 OK. the COVID-19 WhatsApp Chatbot profile contains some of the described components/ functions

4 Accurate. the COVID-19 WhatsApp Chatbot profile contains most of the described components/functions

5 Highly accurate description of the the COVID-19 WhatsApp Chatbot components/functions on profile.

15. Quality of information: Is the COVID-19 WhatsApp Chatbot content correct, well-written, and relevant?

1 Irrelevant/inappropriate/incoherent/incorrect

2 Poor, Barely relevant/appropriate/coherent/may be incorrect

3 Moderately relevant/appropriate/coherent/and appears correct

4 Relevant/appropriate/coherent/correct

5 Highly relevant, appropriate, coherent, and correct

16. Quantity of information: Is the coverage within the COVID-19 WhatsApp Chatbot scope comprehensive and concise?

1 Minimal or overwhelming

2 Insufficient or possibly overwhelming

3 OK but not comprehensive or concise

4 Offers a broad range of information, has some gaps or unnecessary detail; or has no links to more information and resources

5 Comprehensive and concise; contains links to more information and resources

17. Visual information: Is the visual explanation of concepts – through charts/graphs/images/ etc. – clear, logical, correct?

1 Completely unclear/confusing/wrong or necessary but missing

2 Mostly unclear/confusing/wrong

3 OK but often unclear/confusing/wrong

4 Mostly clear/logical/correct with negligible issues

5 Perfectly clear/logical/correct

App subjective quality

SECTION E

20. Would you recommend the COVID-19 WhatsApp Chatbot to people who might benefit from it?

1 Not at all I would not recommend this app to anyone

2 There are very few people I would recommend this app to

3 Maybe There are several people whom I would recommend it to

4 There are many people I would recommend this app to

5 Definitely I would recommend this app to everyone

21. How many times do you think you would usethe COVID-19 WhatsApp Chatbot in the next 12 months if it was relevant to you?

1 None

2 1-2

3 3-10

4 10-50

5 >50

23. What is your overall star rating of thethe COVID-19 WhatsApp Chatbot?

1 ★ One of the worst apps I’ve used

2 ★★

3 ★★★ Average

4 ★★★★

5 ★★★★★ One of the best apps I've used

SECTION F

1. Awareness: Is the COVID-19 WhatsApp Chatbot likely to increase awareness of the importance of addressing COVID-19 myths

Strongly disagree

1 2 3 4

Strongly Agree 5

2. Knowledge: the COVID-19 WhatsApp Chatbot is likely to increase knowledge/understanding of COVID-19 knowledge among the general public and health workers

Strongly disagree

1 2 3 4

Strongly Agree 5

3. Attitudes: The COVID-19 WhatsApp Chatbot is likely to change attitudes toward improving COVID-19 response.

Strongly disagree

1 2 3 4

Strongly Agree 5

4. Intention to change: This app is likely to increase intentions/motivation to address [insert target health behaviour]

Strongly disagree

1 2 3 4

Strongly Agree 5

5. Help-seeking: Use of the COVID-19 WhatsApp Chatbot is likely to encourage the general public to further health-seeking behavior on COVID-19

Strongly disagree

1 2 3 4

Strongly Agree 5

6. Behaviour change: Use of the COVID-19 WhatsApp Chatbot is likely to increase/decrease COVID-19-related healthy behaviours (e.g., wearing of face masks, sanitising, and hand washing)

Strongly disagree

1 2 3 4

Strongly Agree

Annexe 2: Technology Acceptance Model (TAM) Pre-Post Survey Questionnaire-Health Workers

Section Demographic

1.	Age ………………

2.	Sex

●	male

●	female

3.	District …………………. Categorical/ Factor

●	Blantyre

●	Likoma

●	Mzimba South

●	Lilongwe

●	Neno

●	Mzimba North

●	Zomba

●	Mwanza

●	Ntchisi

●	Rumphi

4.	Regional …………….

●	North

●	South

●	Central

5.	Which Pillar did you belong to when supporting the COVID-19 Response? …………

●	Surveillance

●	Laboratory

●	EPI

●	Emergency Operation Centre

●	Risk Communication and Community Engagement,

●	Infection Prevention and Disease Control.

●	Point of Entry.

●	Operation Supply and Logistics (OSL)

●	Digital Health Division

●	CMED/ Coordination and Resource Mobilization

●	Coordination and Resource Mobilizatio

6.	Number of years of experience in COVID-19 response…...Numeric

7.	Occupation (designation)………

●	Surveillance Officer

●	Medical Doctor

●	Nursing Officer

●	Lab Technician

●	Clinician

●	Pharmacists

●	Data Officer

●	Software Developer

●	others specific

Perceived Usefulness of COVID-19 chatbot

8.	The COVID-19 chatbot improved COVID-19 Surveillance

●	Strongly disagree

●	Disagree

●	Neutral

●	Agree

●	Strongly Agree

9.	The COVID-19 chatbot improved awareness of COVID-19 information / Vaccination services

●	Strongly disagree

●	Disagree

●	Neutral

●	Agree

●	Strongly Agree

10.	COVID-19 chatbot increased Surveillance activities, especially case detection and reporting ●	Strongly disagree

●	Disagree

●	Neutral

●	Agree

●	Strongly Agree

11.	COVID-19 chatbot increased awareness of COVID-19 information/ Vaccination services

●	Strongly disagree

●	Disagree

●	Neutral

●	Agree

●	Strongly Agree

12.	The COVID-19 chatbot makes detecting and reporting cases from the community easier.

●	Strongly disagree

●	Disagree

●	Neutral

●	Agree

●	Strongly Agree

13.	The COVID-19 chatbot made it easier to find information on COVID-19 vaccination …

●	Strongly disagree

●	Disagree

●	Neutral

●	Agree

●	Strongly Agree

14.	COVID-19 chatbot enhances COVID-19 surveillance, Health information, and Vaccination services

●	Strongly disagree

●	Disagree

●	Neutral

●	Agree

●	Strongly Agree

Perceived Ease of use of COVID-19 chatbot

15.	I find the COVID-19 chatbot easy to use

●	Strongly disagree

●	Disagree

●	Neutral

●	Agree

●	Strongly Agree

16.	Learning how to use the COVID-19 chatbot is easy for me

●	Strongly disagree

●	Disagree

●	Neutral

●	Agree

●	Strongly Agree

17.	It is easy to become skilful in using the COVID-19 chatbot

●	Strongly disagree

●	Disagree

●	Neutral

●	Agree

●	Strongly Agree

18.	My Interaction with the COVID-19 chatbot was clear

●	Strongly disagree

●	Disagree

●	Neutral

●	Agree

●	Strongly Agree

19.	My Interaction with the COVID-19 chatbot was understandable

●	Strongly disagree

●	Disagree

●	Neutral

●	Agree

●	Strongly Agree

20.	It will be easy for me to find information through the COVID-19 chatbot

●	Strongly disagree

●	Disagree

●	Neutral

●	Agree

●	Strongly Agree

21.	Are COVID-19 chatbot features easy to use and follow through?

●	Strongly disagree

●	Disagree

●	Neutral

●	Agree

●	Strongly Agree

Attitude of COVID-19 chatbot

22.	Conducting surveillance using the COVID-19 chatbot was an excellent idea

●	Strongly disagree

●	Disagree

●	Neutral

●	Agree

●	Strongly Agree

23.	Sharing COVID-19 information/ Vaccination services was a good idea

●	Strongly disagree

●	Disagree

●	Neutral

●	Agree

●	Strongly Agree

24.	I am confident about using the COVID-19 chatbot for COVID-19 surveillance and COVID-19 vaccination services.

●	Strongly disagree

●	Disagree

●	Neutral

●	Agree

●	Strongly Agree

25.	The COVID-19 chatbot helps to increase engagement in COVID-19 surveillance and vaccination services.

●	Strongly disagree

●	Disagree

●	Neutral

●	Agree

●	Strongly Agree

26.	I prefer using a COVID-19 chatbot for COVID-19 Surveillance and COVID-19 vaccination services.

●	Strongly disagree

●	Disagree

●	Neutral

●	Agree

●	Strongly Agree

27.	I believe it is a good idea to use a COVID-19 chatbot for future COVID-19 surveillance

●	Strongly disagree

●	Disagree

●	Neutral

●	Agree

●	Strongly Agree

Behavioural Intention to use chatbot Technology for any Pandemic and Epidemic

28.	I intend to use the mobile chatbot for surveillance frequently.

●	Strongly disagree

●	Disagree

●	Neutral

●	Agree

●	Strongly Agree

29.	I intend to use the chatbot technology for epidemic heavily

●	Strongly disagree

●	Disagree

●	Neutral

●	Agree

●	Strongly Agree

30.	I intend to use the chatbot technology for future outbreaks

●	Strongly disagree

●	Disagree

●	Neutral

●	Agree

●	Strongly Agree

31.	I intend to use the repetitive chatbot technology for epidemics as often as possible.

●	Strongly disagree

●	Disagree

●	Neutral

●	Agree

●	Strongly Agree

Addition Question

32.	How long have you used the COVID-19 chatbot

33.	Would you use it again?

●	Yes

●	No

34.	If Yes/No, give reasons why to use it again

35.	What additional features would you like to see in the COVID-19 chatbot

36.	What features would you like to remove from the COVID-19 chatbot

Annexes 3: Pre-Post Survey Questionnaire -General Public

Section Demographic

1.	Age ………………

2.	Sex ………………….

●	Male

●	female

3.	District ………………….

●	Blantyre

●	Likoma

●	Mzimba South

●	Lilongwe

●	Neno

●	Mzimba North

●	Zomba

●	Mwanza

●	Ntchisi

●	Rumphi

4.	Regional

●	North

●	South

●	Central

5.	Occupation (designation)

●	Farmer

●	Fisherman

●	Fish preparation /Fish seller

●	Business, seller, trader

●	Housewife

●	Civil servant (teacher, police…)

●	Student

●	Retired

●	Other, detail

Perceived Usefulness of COVID-19 chatbot

6.	The COVID-19 chatbot improved awareness of COVID-19 Information, including Vaccination statistics.

●	Strongly disagree

●	Disagree

●	Neutral

●	Agree

●	Strongly Agree

7.	COVID-19 chatbot increased awareness of COVID-19 information and vaccination services

●	Strongly disagree

●	Disagree

●	Neutral

●	Agree

●	Strongly Agree

8.	The COVID-19 chatbot makes noticing and reporting COVID-19 suspects from the community easier.

●	Strongly disagree

●	Disagree

●	Neutral

●	Agree

●	Strongly Agree

9.	The COVID-19 chatbot made it easier to find information on COVID-19 vaccination statistics.

●	Strongly disagree

●	Disagree

●	Neutral

●	Agree

●	Strongly Agree

10.	COVID-19 chatbot enhances the effectiveness of COVID-19 surveillance and COVID-19 information and Vaccination

●	Strongly disagree

●	Disagree

●	Neutral

●	Agree

●	Strongly Agree

Perceived Ease of use of COVID-19 chatbot

11.	I find the COVID-19 chatbot easy to use

●	Strongly disagree

●	Disagree

●	Neutral

●	Agree

●	Strongly Agree

12.	Learning how to use the COVID-19 chatbot is easy for me

●	Strongly disagree

●	Disagree

●	Neutral

●	Agree

●	Strongly Agree

13.	It is easy to become skillful in using the COVID-19 chatbot

●	Strongly disagree

●	Disagree

●	Neutral

●	Agree

●	Strongly Agree

14.	My Interaction with the COVID-19 chatbot was clear

●	Strongly disagree

●	Disagree

●	Neutral

●	Agree

●	Strongly Agree

15.	My Interaction with the COVID-19 chatbot was understandable

●	Strongly disagree

●	Disagree

●	Neutral

●	Agree

●	Strongly Agree

16.	It will be easy for me to find information through the COVID-19 chatbot

●	Strongly disagree

●	Disagree

●	Neutral

●	Agree

●	Strongly Agree

17.	Are COVID-19 chatbot features easy to use and follow through?

●	Strongly disagree

●	Disagree

●	Neutral

●	Agree

●	Strongly Agree

Attitude of COVID-19 chatbot

18.	Reporting COVID-19 suspects using the COVID-19 chatbot was an excellent idea

●	Strongly disagree

●	Disagree

●	Neutral

●	Agree

●	Strongly Agree

19.	Sharing COVID-19 information/ Vaccination services was a good idea

●	Strongly disagree

●	Disagree

●	Neutral

●	Agree

●	Strongly Agree

20.	I am confident about using the COVID-19 chatbot for COVID-19 reporting and COVID-19 vaccination services.

●	Strongly disagree

●	Disagree

●	Neutral

●	Agree

●	Strongly Agree

21.	The COVID-19 chatbot helps to increase engagement in COVID-19 reporting of suspects from the community and vaccination services.

●	Strongly disagree

●	Disagree

●	Neutral

●	Agree

●	Strongly Agree

22.	I prefer using COVID-19 chatbots for COVID-19 reporting of suspected cases from the community and COVID-19 vaccination services.

●	Strongly disagree

●	Disagree

●	Neutral

●	Agree

●	Strongly Agree

23.	I believe it is a good idea to use chatbot technology in the future Pandemic/Epidemic Response

●	Strongly disagree

●	Disagree

●	Neutral

●	Agree

●	Strongly Agree

Behavioural Intention to use of COVID-19 chatbot

24.	I intend to frequently use the mobile COVID-19 chatbot for COVID-19 reporting suspected cases from the community and COVID-19 vaccination.

●	Strongly disagree

●	Disagree

●	Neutral

●	Agree

●	Strongly Agree

25.	I intend to use the COVID-19 chatbot heavily

●	Strongly disagree

●	Disagree

●	Neutral

●	Agree

●	Strongly Agree

26.	I intend to use the COVID-19 chatbot outbreak

●	Strongly disagree

●	Disagree

●	Neutral

●	Agree

●	Strongly Agree

27.	I intend to use the repetitive COVID-19 chatbot as often as possible.

●	Strongly disagree

●	Disagree

●	Neutral

●	Agree

●	Strongly Agree

Addition Question

28.	How long have you used the COVID-19 chatbot

29.	Would you use it again?

●	Yes

●	No

30.	If Yes/No, give reasons why to use it again

31.	What additional features would you like to see in the COVID-19 chatbot

32.	What features would you like to remove from the COVID-19 chatbot

Annex 4 National Workshop Group Discussion – Guiding Questions

Perceived Usefulness of COVID-19 chatbot

1.	How did the COVID-19 chatbot improve COVID-19 Surveillance

2.	How did the COVID-19 chatbot improve awareness of COVID-19 information / Vaccination services

3.	Do you think the COVID-19 chatbot increases Surveillance activities, especially case detection and reporting

4.	If No/ Yes How?

5.	Do you think the COVID-19 chatbot increased awareness of COVID-19 information/ Vaccination services…..HOW?

6.	How did The COVID-19 chatbot make detecting and reporting cases from the community easier?

7.	How did The COVID-19 chatbot make finding information on COVID-19 vaccination easier?

8.	How did the COVID-19 chatbot enhance the effectiveness of COVID-19 surveillance and COVID-19 information and Vaccination?

Perceived Ease of use of COVID-19 chatbot

9.	Do you think the COVID-19 chatbot is easy to use ………… HOW?

10.	Do you think learning how to use a COVID-19 chatbot is easy………… HOW?

11.	Do you think it is easy to become skilful in using the COVID-19 ………… HOW?

12.	Do you think Interaction with the COVID-19 chatbot was clear……HOW?

13.	Do you think Interaction with the COVID-19 chatbot was understandable……HOW?

14.	Is it easy to find COVID-19 information through the COVID-19 chatbot..HOW?

15.	Are COVID-19 chatbot features easy to use and follow through? …………HOW?

Attitude of COVID-19 chatbot

16.	How is conducting surveillance using the COVID-19 chatbot a good idea…………

17.	How is sharing COVID-19 information/ Vaccination services a good idea

18.	The group has agreed that it feels positive towards using the COVID-19 chatbot for COVID-19 surveillance and COVID-19 vaccination services.

19.	How does the COVID-19 chatbot help to increase engagement in COVID-19 surveillance and vaccination services?

20.	The group has concluded that it favours using the COVID-19 chatbot for COVID-19 Surveillance and COVID-19 vaccination services.

21.	The group has concluded that using a COVID-19 chatbot for future COVID-19 surveillance is a good idea.

Behavioural Intention to use of COVID-19 chatbot

22.	The group has concluded that they intend to frequently use the mobile COVID-19 chatbot for COVID-19 surveillance and COVID-19 vaccination.

23.	The group has concluded it intends to use the COVID-19 chatbot heavily

24.	The group has concluded to wants to use the COVID-19 chatbot outbreak

25.	The group has decided to use the repetitive COVID-19 chatbot as often as possible.
